# Education, Health Literacy, and Burden Among Dementia Care Partners at Hospital Discharge

**DOI:** 10.1093/geroni/igaf122.1957

**Published:** 2025-12-31

**Authors:** Ashley Kuzmik

**Affiliations:** Alzheimer’s Association, Chicago, Illinois, United States

## Abstract

Care partners of hospitalized persons with dementia often experience significant burden at the time of discharge. This study examined whether dementia care partner health literacy mediates the relationship between education and burden at hospital discharge. Data from 277 care partners in the Family-centered Function-focused Care (Fam-FFC) trial were analyzed using mediation analysis to assess indirect effects of education on burden (Short-Form Zarit Burden Interview [ZBI-12]) through health literacy (Rapid Estimate of Adult Literacy in Medicine-Short Form [REALM-SF]). Care partners had an average age of 60.4 years (SD = 14.3), with 71.8% identifying as female and the majority being White (53.1%) or Black (43.7%). Education levels were categorized as follows: 8.3% had low education (less than high school), 53.1% had medium education (high school, some college, or trade school), and 38.6% had high education (college degree or above). For medium education (B = -0.012; 95% CI = -0.019, -0.003), the indirect effect accounted for 18.5% of the total effect, while for high education (B = -0.018; 95% CI = -0.026, -0.006), it accounted for 21.4%, indicating partial mediation. Findings underscore the role of health literacy in reducing care partner burden and highlight the need to address education and literacy disparities to provide effective support at hospital discharge.

